# GermlncRNA: a unique catalogue of long non-coding RNAs and associated regulations in male germ cell development

**DOI:** 10.1093/database/bav044

**Published:** 2015-05-16

**Authors:** Alfred Chun-Shui Luk, Huayan Gao, Sizhe Xiao, Jinyue Liao, Daxi Wang, Jiajie Tu, Owen M. Rennert, Wai-Yee Chan, Tin-Lap Lee

**Affiliations:** ^1^Reproduction, Development and Endocrinology Program, School of Biomedical Sciences, Faculty of Medicine, ^2^The Chinese University of Hong Kong-Shandong University (CUHK-SDU) Joint Laboratory on Reproductive Genetics and ^3^ CUHK-BGI Innovation Institute of Trans-Omics, The Chinese University of Hong Kong, Shatin, Hong Kong, China, ^4^GigaScience, Beijing Genomics Institute-Hong Kong (BGI-HK) Research Institute, 16 Dai Fu Street, Tai Po Industrial Estate, Hong Kong, China, ^5^Beijing Genomics Institute-Shenzhen (BGI-SZ), Beishan Industrial Zone, Yantian District, Shenzhen, China and ^6^The Eunice Kennedy Shriver National Institute of Child Health and Human Development, National Institutes of Health, Bethesda, MD, USA

## Abstract

Spermatogenic failure is a major cause of male infertility, which affects millions of couples worldwide. Recent discovery of long non-coding RNAs (lncRNAs) as critical regulators in normal and disease development provides new clues for delineating the molecular regulation in male germ cell development. However, few functional lncRNAs have been characterized to date. A major limitation in studying lncRNA in male germ cell development is the absence of germ cell-specific lncRNA annotation. Current lncRNA annotations are assembled by transcriptome data from heterogeneous tissue sources; specific germ cell transcript information of various developmental stages is therefore under-represented, which may lead to biased prediction or fail to identity important germ cell-specific lncRNAs. GermlncRNA provides the first comprehensive web-based and open-access lncRNA catalogue for three key male germ cell stages, including type A spermatogonia, pachytene spermatocytes and round spermatids. This information has been developed by integrating male germ transcriptome resources derived from RNA-Seq, tiling microarray and GermSAGE. Characterizations on lncRNA-associated regulatory features, potential coding gene and microRNA targets are also provided. Search results from GermlncRNA can be exported to Galaxy for downstream analysis or downloaded locally. Taken together, GermlncRNA offers a new avenue to better understand the role of lncRNAs and associated targets during spermatogenesis.

Database URL: http://germlncrna.cbiit.cuhk.edu.hk/

## Introduction

Male infertility accounts for more than half of the diagnosed infertility cases worldwide (1, 2). Though the unique cellular dynamics of germ cell development provides a representative model for understanding the fundamentals of developmental biology, our current understanding of the molecular mechanisms in male germ cell development remains largely elusive. This poses significant challenges on the effective development of therapeutic regimen and clinical management.

Spermatogenesis refers to the continuous multi-stage processes by which spermatogonial stem cells on the seminiferous tubular basement membrane proliferate and differentiate into subsequent cellular stages, including spermatogonia (Spga), spermatocytes (Spcy) and spermatids (Sptd), and finally to functional spermatozoa which are released into the seminiferous tubule lumen. Successful spermatogenesis relies on the precise transcriptional programs. To identify the regulatory networks involved in male germ cell development, we previously applied serial analysis of gene expression (SAGE) and developed GermSAGE (3) and GonadSAGE (4) databases. We identified a number of gene networks associated with stage-specific transcription factors (TFs) and promoter elements. Importantly, >45% transcripts were unannotated (3, 5–9), suggesting many novel transcripts and corresponding functions remain to be explored. Importantly, many of them were suggested to be non-coding RNAs (6, 9).

Recently, long non-coding RNAs (lncRNAs) were widely identified as novel regulators in normal and disease development (10–16). Unlike small RNAs like piwi-interacting RNA (piRNA) or microRNA, the regulatory roles of lncRNAs are poorly defined. Recent studies demonstrated lncRNAs exert activating or inhibitory regulation through interaction with mRNA (17), DNA (18), microRNA (19), histone modifier (20), RNA-binding protein (21) and chromatin (22, 23). Presently it is estimated that more than 40 000 unique lncRNAs are expressed in the mammalian cells (16). Recent studies of the role of lncRNAs in mammalian testis development and spermatogenesis suggested lncRNAs are dynamically regulated (24, 25). Expression profiling analyses on primordial germ cell reprogramming and postnatal germ cell development have revealed that thousands of lncRNAs are significantly altered and correlated with nearby mRNA gene clusters (24). Comparison on neonatal and adult mouse testes has also demonstrated dynamic lncRNA expression and exhibited associations with epigenetic modifications and evolutionary conserved elements (26). Among the major male germ cell stages in spermatogenesis, type A spermatogonia shows the maximum number of lncRNA candidates (25). This is concordant with the expression pattern of mRNAs.

Though lncRNA research in male germ cell development presently exhibits momentum, only few functional lncRNAs in spermatogenesis such as *Tsx*, *HongrES2*, *Mrhl* and *Spga-lncRNA* have been reported (13). To systematically identify and predict functional lncRNAs, the knowledge of lncRNA annotation is a prerequisite. Although lncRNA annotations are publicly available in genomic databases like Ensembl and NONCODE (27, 28), the transcripts are derived from expression data from major tissues and cell types. As the expression profile of lncRNAs was reported to be tissue- or cell-specific (29–32). This partly explains why only few lncRNAs were identified in male germ cell development to date (13). Here we hypothesize that male germ cell-specific lncRNAs are under-represented in the current public annotations, leading to biased lncRNA predictions or failure to detect critical lncRNAs in specialized cell type during male germ development.

In this study, we report the construction of GermlncRNA, a specialized lncRNA database of male germ cell development by assembling male germ cell transcriptome evidence from three different expression platforms. In addition to conventional lncRNA annotations, we also include novel male germ cell-specific lncRNAs derived from *de novo* analysis of transcriptome data, and provide comprehensive regulatory and functional prediction information, which offers a new avenue to better understand on the role of lncRNAs and associated targets in spermatogenesis.

## Materials and methods

### Database construction and structure

GermlncRNA is an open-access database developed on Linux using SQlite platform with a Personal Home Page-based Yii framework to store, retrieve and exchange all acquired data.GermlncRNA was developed based on transcriptomic and regulatory data from our previous studies and public domain data. An overview of data organization in GermlncRNA is shown in a schema diagram (Supplementary Figure S1). The lncRNA annotations of Ensembl (release 74), RefSeq (NCBI37/mm9) and University of California Santa Cruz (UCSC) Genes (mm9) were obtained from the UCSC Table Browser (http://genome.ucsc.edu/cgi-bin/hgTables). Annotations from NONCODEv4 and fRNAdb were downloaded from http://www.noncode.org and http://www.ncrna.org/frnadb, respectively. Information on regulatory elements [polyadenylation, cap analysis of gene expression (CAGE), DNase hypersensitive sites, histone modifications and conserved elements] were obtained from corresponding data tracks in UCSC Genome Browser (http://genome.ucsc.edu/index.html). Experimentally validated TF binding data in mouse genome were retrieved from ChIPBase (http://deepbase.sysu.edu.cn/chipbase/lncrna.php). For expression data, RNA-Seq data were retrieved from European nucleotide archive (http://www.ebi.ac.uk/ena) (for germ cells) and NONCODEv4 (http://www.noncode.org) (for other tissues: heart, hippocampus, liver, lung, spleen and thymus), while tiling and gene expression microarrays data were from gene expression omnibus (http://www.ncbi.nlm.nih.gov/geo). SAGE datasets were retrieved from GermSAGE (http://germsage.nichd.nih.gov/germsage/home.html).

### *lncRNA prediction in male germ cell development*

To identify unique lncRNAs expressed in male germ cell development, we developed a bioinformatics pipeline known as hybrid transcriptome assembly (HTA, Supplementary Figure S2) to integrate the polyadenylated germ cell transcriptomes from GermSAGE (3) and tiling microarray (33) previously published by us, together with two recent RNA sequencing (RNA-Seq) datasets (34, 35). In brief, raw reads from RNASeq studies SRP018124 and SRP010262 (34, 35) were aligned to mm9 mouse genome and assembled by TopHat-Cufflinks with *de novo* approach (36), while tiling array data in cell intensity file format (GSE55318) were processed by Affymetrix Tiling Array Software using Affymetrix binary probe mapping genomic location files version 35 as a reference to identify transcribed fragments known as transfrags with bandwidth 75 bp ‘One-side Upper’ test (33). The genomic loci covered by both platforms were retained and were subsequently classified into annotated or unannotated categories. To generate a complete catalogue of annotated lncRNAs, annotations from five public databases, including Ensembl (28), RefSeq (37), UCSC Genes (38), NONCODE (27) and fRNAdb (39) were obtained in browser extensible data or gene transfer format files. The sequence of each annotated lncRNA was retrieved using Galaxy based on these annotations, and the lncRNA candidates with the identical sequence and genomic locus were regarded as the same transcript. The lncRNA transcripts not covered by any of the five reference public databases were identified as *de novo* putative lncRNAs. To reduce ambiguities on lncRNA prediction, all *de novo* putative lncRNAs overlapping coding genes were ignored. Only intergenic lncRNAs predicted to be non-coding by coding-potential assessment tool (40) were selected. These lncRNA candidates were referred to as ‘novel’ lncRNAs. Transcription evidence of both annotated and novel lncRNAs was further evaluated by searching for the presence of SAGE tags near the 3′-terminal from GermSAGE. To give a better picture on prediction quality, the lncRNAs were categorized into four classes according to the number (from 1 to 4) of expression evidence (Table 1). Each lncRNA candidate in GermlncRNA was assigned with a unique GermlncRNA identity (ID). In GermlncRNA database, the expression of lncRNAs was presented in fragments per kilobase of exon per million fragments mapped (FPKM) by RNASeq or tag counts (with tag sequence) by GermSAGE. We defined the RNA-seq thresholds for detectable and abundant expressions as 0.3 and 3 FPKM, respectively (41). SAGE tag of an lncRNA represents the 10 bp sequence downstream to the 3′ most enzymatic restriction site CATG (42). The tag count represents the expression level of an lncRNA in the corresponding stage. Transcripts that showed more than one SAGE tag were considered expressed, whereas transcripts showed more than five tag counts were considered abundantly expressed. The normalized expression data from the whole testes of neonatal (6 day) and adult (8 week) mice were also included (GSE43442) (26) for comparison.

**Table 1.** Data content and distribution of GermlncRNA

Category of lncRNA	Number (%)
Annotated lncRNA	
*By type*	
Intergenic	36 298 (32.9%)
Exonic	49 679 (45.0%)
Intronic	19 646 (17.8%)
Promoter-associated	4853 (4.4%)
*By expression*	
≥0.3 FPKM at	
Any stage	43 215 (39.1%)
Spermatogonia	25 322 (22.9%)
Spermatocytes	29 789 (27.0%)
Spermatids	28 050 (25.4%)
≥3 FPKM at	
Any stage	18 409 (16.7%)
Spermatogonia	8923 (8.1%)
Spermatocytes	11 199 (10.1%)
Spermatids	11 587 (10.5%)
Absence of SAGE tag	7832 (7.1%)
Tag count ≥1 at	
Any stage	34 529 (31.3%)
Spermatogonia	18 958 (17.2%)
Spermatocytes	20 942 (19.0%)
Spermatids	19 520 (17.7%)
Tag count ≥5 at any stage	
Any stage	8574 (7.8%)
Spermatogonia	5360 (4.9%)
Spermatocytes	5448 (4.9%)
Spermatids	5180 (4.7%)
Novel (intergenic) lncRNA	
*By type*	
Intergenic	2357 (84.5%)
Promoter-associated	433 (15.5%)
*By expression*	
≥0.3 FPKM at	
Any stage	2786 (99.9%)
Spermatogonia	1009 (36.2%)
Spermatocytes	2206 (79.1%)
Spermatids	2532 (90.8%)
≥3 FPKM at	
Any stage	2059 (73.8%)
Spermatogonia	710 (25.4%)
Spermatocytes	1259 (45.1%)
Spermatids	1560 (55.9%)
Absence of SAGE tag	337 (12.1%)
Tag count ≥1 at	
Any stage	812 (29.1%)
Spermatogonia	410 (14.7%)
Spermatocytes	494 (17.7%)
Spermatids	432 (15.5%)
Tag count ≥5 at	
Any stage	187 (6.7%)
Spermatogonia	119 (4.3%)
Spermatocytes	115 (4.1%)
Spermatids	108 (3.9%)
*By evidence level*	
Class 1 (1 RNASeq + Tiling arrays)	1155 (41.4%)
Class 2 (2 RNASeq + Tiling arrays)	934 (33.5%)
Class 3 (1 RNASeq + Tiling arrays + SAGE)	344 (12.3%)
Class 4 (2 RNASeq + Tiling arrays + SAGE)	357 (12.8%)

### Association of regulatory features 

Genomic data on regulatory features, including polyadenylation (PolyA), DNase hypersensitive sites, CAGE, histone modifications (testis) and conserved elements were all retrieved from the corresponding data tracks in UCSC Genome Browser (Table 2). To reveal the potential association of a regulatory feature with lncRNA, the number of the corresponding feature in the promoter, gene body or around 5′/3′-terminal region of lncRNAs was counted. The percentage of association with each feature for annotated and novel lncRNAs was calculated and presented in a bar chart, while the significance of difference between annotated and novel lncRNAs was assessed and compared by two-sided Chi-square test.

**Table 2.** List of genomic features in GermlncRNA

Symbol	Full name	Definition of positive association	Biological implication
PolyA	Polyadenylation	Any overlap from − 50 to + 200 bp relative to transcription termination sites (TTS)	3′-Terminal for a transcript
CAGE	Cap analysis of gene expression	Any overlap from −200 to + 50 bp relative to TSS	5′-Terminal for a transcript
DHS	DNase I hypersensitivity site	Significant overlap (≥10 bp) of strong (top 50%) DHS signals with promoters (upstream 2000 bp)	More accessible to DNA-binding proteins, such as TFs and regulators
H3K4me1	Histone 3 mono-methylated lysine 4	Significant overlap of strong H3K4me1 signal with promoters or gene bodies (from TSS to TTS)	Enriched in enhancers
H3K4me3	Histone 3 tri-methylated lysine 4	Significant overlap of strong H3K4me3 signal with promoters	Enriched in active promoters
H3K27ac	Histone 3 acetylated lysine 27	Significant overlap of strong H3K27ac signal with promoters	Enriched in active promoters
H3K27me3	Histone 3 tri-methylated lysine 27	Significant overlap of strong H3K27me3 signal with promoters	Enriched in repressed promoters
H3K36me3	Histone 3 tri-methylated lysine 36	Significant overlap of strong H3K36me3 signal with promoters or gene bodies	Enriched in actively transcribed regions
Conservedelements	PhastCons placental mammal-conserved elements (30-way Multiz alignment)	Significant overlap of PhastCons placental mammal-conserved elements with gene bodies	Conserved regions among mammals

### GermlncRNA function prediction

In GermlncRNA, the protein-coding transcripts next to novel lncRNAs in either direction were classified as *cis*-regulation category. Proximal coding gene neighbours of a novel lncRNA were identified from RefSeq gene annotation, and the distance between each novel lncRNA and its *cis*-acting target was calculated. Sequence homology between novel lncRNAs and coding mRNAs were predicted by basic local alignment search tool (BLAST) (43). lncRNAs that showed significant similarity to coding gene sequences on the other chromosomes (at least 85% within 200 bp) were classified as *trans*-acting. The gene ontologies of *cis*- and *trans*-associated gene were retrieved from The Database for Annotation, Visualization and Integrated Discovery (http://david.abcc.ncifcrf.gov/) (44, 45).

### MicroRNA target prediction

Potential microRNA (miRNA) targets on the novel lncRNAs were identified by Probability of Interaction by Target Accessibility (PITA) algorithm (http://genie.weizmann.ac.il/pubs/mir07/index.html) (46), which predicts the microRNA-target interaction by a free energy change (ΔΔG) approach. In brief, full-length novel lncRNA sequences in fasta format were scanned for the presence of miRNA response elements (MREs). To identify significant miRNA interaction, the results were filtered by ΔΔG cut-off of below −10. The representative miRNA targets for each novel lncRNA are listed in the search result table when ‘predicted microRNA targets’ is selected, and the complete details on miRNA candidates can be retrieved by clicking on the representative miRNAs.

### Transcriptional regulation

To identify potential transcriptional regulation on the annotated lncRNAs, experimentally supported TFs binding information based on chromatin immunoprecipitation-sequencing (ChIP-Seq) were retrieved from ChIPBase (47), with a focus on promoter interactions. Such information was then mapped to the annotated lncRNAs based on similar identifiers. The information was saved in ‘ChIPBase’ column.

## Results

### Database overview

**Figure 1.**
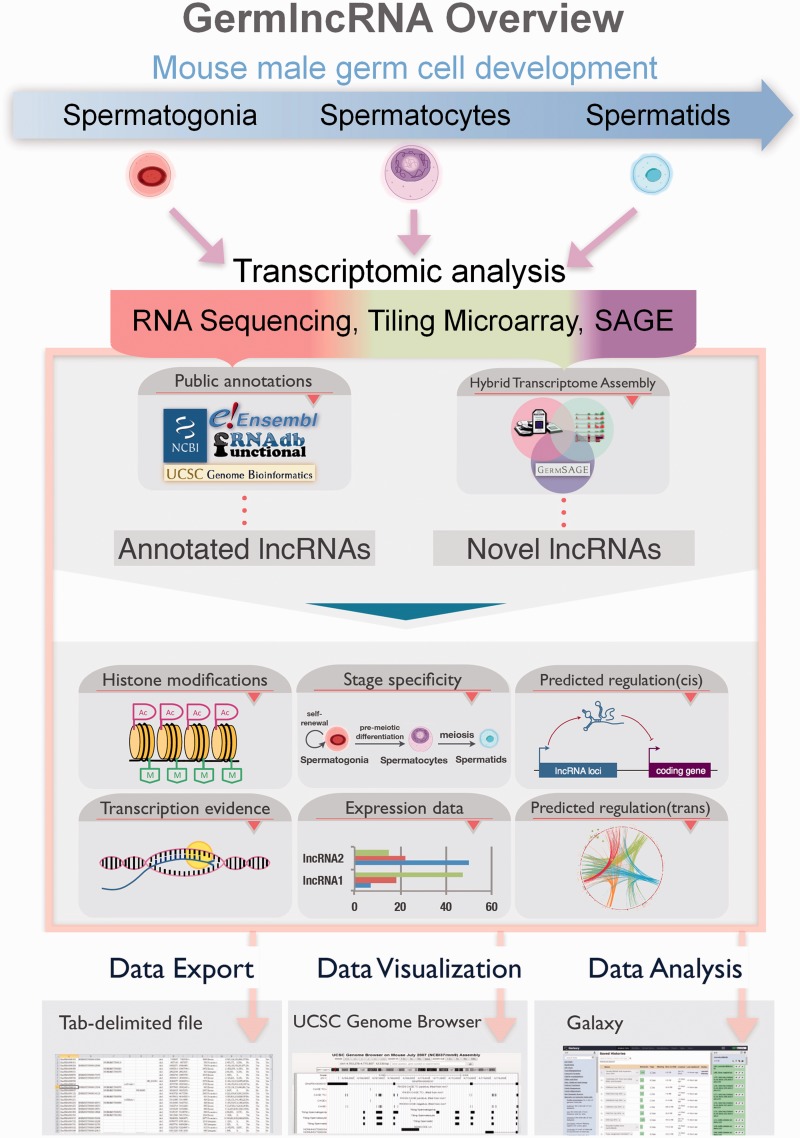
GermlncRNA overview. To study lncRNA biology in mouse germ cell development, we made use of high-throughput transcriptomic data on three germ cell stages from three platforms, namely RNA sequencing, tiling microarray and SAGE, and identified germ cell-specific novel lncRNAs. Annotations from five public databases, including Ensembl, RefSeq, UCSC Genes, Non-code and fRNAdb were combined to obtain a catalogue of annotated lncRNAs. Both annotated and novel lncRNAs were analysed for expression, association with regulatory features and functional implications. The search results in GermlncRNA can be exported as text file, visualized and further analysed in Galaxy.

**Figure 2.**
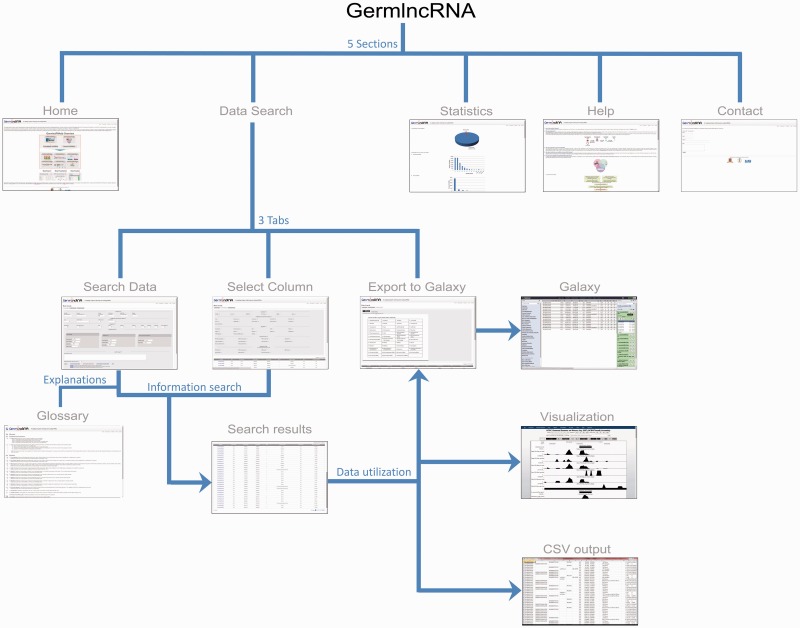
GermlncRNA structure. The database consists of five main sections: Home, Data Search, Statistics, Help and Contact. The Data Search section provides the core lncRNA information by the help of three tabs—Search data, Select column and Export to Galaxy (48). A Glossary panel provides explanations for key terms in Data Search sections. Furthermore, the lncRNAs in search results can be visualized in UCSC Genome Browser or downloaded in a text (CSV) file.

GermlncRNA database is the first comprehensive lncRNA catalogue on the key stages of male germ cell development, including type A spermatogonia (Spga), pachytene spermatocytes (Spcy) and round spermatids (Sptd). It also provides comprehensive information on regulatory signatures, expression and associated coding genes through *cis-* or *trans-* regulation. The data retrieved from GermlncRNA can be downloaded or further analysed by Galaxy (Figure 1). GermlncRNA is divided into five sections, including home, data search panel, statistics, help and contact (Figure 2).

#### Home

The home section is the front page of GermlncRNA, where it provides basic background information about GermlncRNA and the database overview. This section is updated regularly to highlight any changes or new functions implemented in GermlncRNA.

#### Data search panel

The search panel is the core element of GermlncRNA that provides a query interface featured with various search options, predefined examples for quick glimpse on GermlncRNA operation (Supplementary Figure S3). It is developed and optimized to be fast, intuitive and functional. To provide flexible data search, selection and export, this section is further divided into three sub-sections in form of tabs located on top of data search interface. A glossary panel for all the terms can be retrieved by clicking on ‘?’ icons in ‘Search Data’ and ‘Select Column’ tabs for quick explanations on key terms.

##### Search data tab

Users can search for lncRNAs of interest through a single or combined selection on the four major search categories, including (i) Annotation IDs from the public databases (Ensembl, RefSeq, UCSC, NONCODE and fRNAdb), (ii) LncRNA type and genomic features (annotated/novel, associated genomic feature, evidence level and chromosome location), (iii) Regulatory signals (Poly A, DNase I hypersensitivity sites (DHS), CAGE, Histone modifications and presence of conserved elements), (iv) Expression data from RNA-seq, SAGE and microarray platforms and (v) Sequence homology search on GermlncRNA candidates by BLAST program version 2.2.30+ (43). Three search examples are provided for user’s reference. Clicking on the example links will show the corresponding selections. The lncRNAs in search result can be visualized in UCSC Genome Browser by clicking on the GermlncRNA ID, downloaded in comma separated values (csv) text format or exported to Galaxy (48) for further analysis by selecting ‘Export to Galaxy’ tab.

##### Select column tab

To provide flexible data output format, the result table column can be further refined before download or export step. The select column page allows users to select what particular features to be included in the data table. When a feature is checked, the data table will be updated in real time accordingly.

##### Export to Galaxy tab

In addition to data retrieval, GermlncRNA also provide analysis option through Galaxy (48), a web tool that provides common genomic analyses in form of workflows. As no programming is required, it is highly accessible to scientists that do not have bioinformatics background. Details on Galaxy can be found here: https://usegalaxy.org/.

#### Statistics

Data statistics on lncRNA composition, distribution of exon number per lncRNA, size and chromosomes, lncRNA types, annotation sources and *cis*/*trans*-regulatory genes are illustrated in graphical format.

#### Help

The Help section contains the details on common questions related to GermlncRNA, data sources applied and supporting references.

#### Contact

Questions, suggestions or comments can be sent to our research team with the aid of online form in this section. We will address every comment promptly.

### Annotated and novel lncRNA catalogues

**Figure 3.**
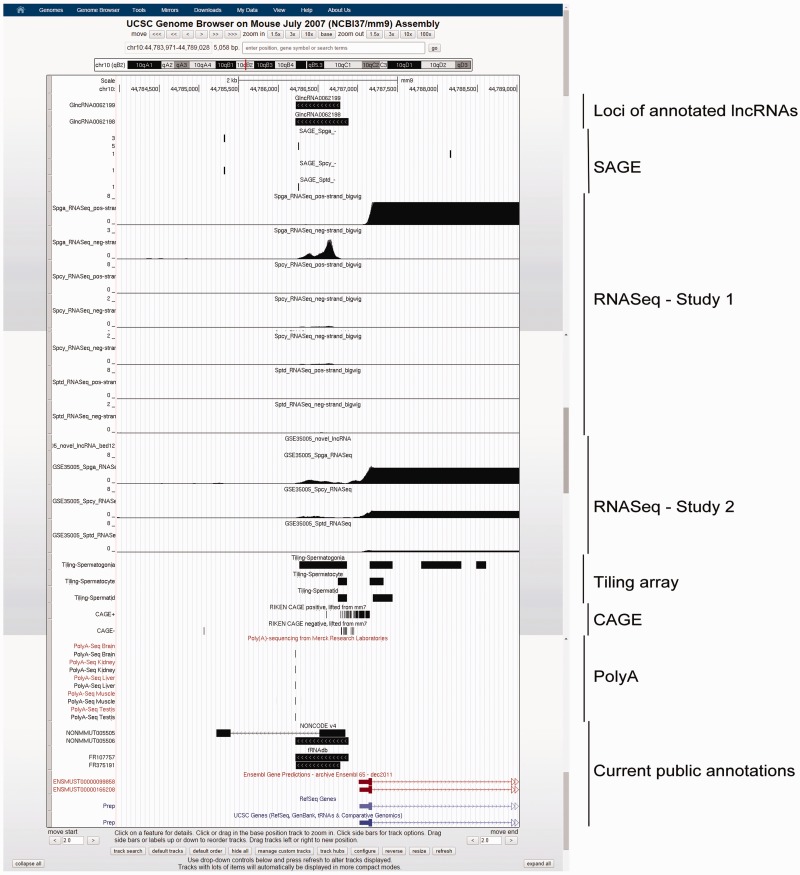
Example of two annotated lncRNAs with similar loci, GlncRNA0062198 and GlncRNA0062199, as viewed in UCSC Genome Browser. As shown, the two lncRNAs have annotated loci of 3′-terminals differ by 2 bp and those of 5′-terminal by 104 bp. The former is annotated by NONCODE and fRNAdb, while the latter is annotated by fRNAdb only. Both of the lncRNAs were supported by Spga-specific SAGE, RNASeq and tiling array evidence, indicating a strong expression specifically in Spga. They are also covered by probes in lncRNA microarray and showed a stronger expression in neonatal testis than in adult testis. Furthermore, the expression is also supported by signals in PolyA sequencing data. They are located in the promoter region of a bidirectional protein-coding gene prolyl endopeptidase (Prep), whose expression is also Spga-specific, suggesting possible regulation in *cis*.

**Figure 4.**
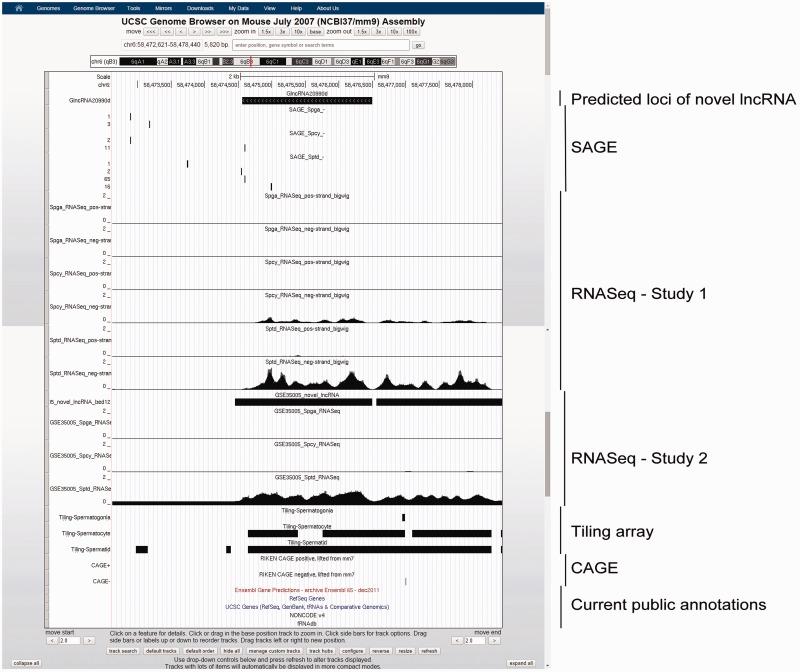
Example of a novel lncRNA, GlncRNA20990d, as viewed in UCSC Genome Browser. This lncRNA is intergenic and previously unannotated by any of the five public genomic databases. It has a gradually increasing expression along spermatogenesis, with specifically highest expression in Sptd, as supported by both SAGE and RNASeq data. A nearby upstream CAGE signal suggested the location of its 5′-terminal.

GermlncRNA database contains a total of 110 476 annotated lncRNAs and 2790 novel intergenic lncRNAs (or simply novel lncRNA in this article) expressed in male germ cell development. Table 1 summarizes the distribution of annotated and novel lncRNAs classified by type of genomic features, expression and evidence level. For annotated lncRNA populations, the RNA size ranged from 200 to 100 989bp with an average of 1628 bp. In total, 62.8% (45.0 + 17.8%) were gene-associated, in which 71.7 and 28.3% of gene-associated candidates were associated with the exons and the introns, respectively. Besides, 32.9% were intergenic, and 4.4% were associated with promoters [2 kb upstream to transcription start sites (TSS) of RefSeq coding genes]. Two examples of annotated promoter-associated lncRNAs are illustrated (Figure 3). The size of novel lncRNAs ranged from 200 to 14 945bp, with an average of 800 bp. In total, 15.5% were located at the promoters of protein-coding genes. Because no supported mRNA evidence was reported previously, the novel lncRNAs may represent male germ cell specific lncRNAs. A total of 357 novel lncRNAs belonged to class four novel lncRNAs (Table 1), which were supported by all three transcriptome sources. A class four novel lncRNA example is shown (Figure 4).

### lncRNA expression analysis

In addition to genomic location, we also examined the expression pattern of lncRNAs. Based on FPKM measurement, 73.9% (2059/2786) of expressed novel lncRNAs and 42.6% (18409/43215) of expressed annotated lncRNAs were highly expressed at all three male germ cell stage (Table 1). Importantly, the percentage of highly expressed lncRNA candidates at every stage in expressed candidates for novel lncRNA group was higher than the annotated group at all the germ cell stages [Spga: 25.5% (710/2786) vs. 20.6% (8923/43 215); Spcy: 45.2% (1259/2786) vs. 25.9% (11 199/43 215); Sptd: 56.0% (1560/2786) vs. 26.8% (11 587/43 215)]. In addition, we also detected a higher percentage of stage-specific lncRNA candidates in the novel group rather than in the annotated group at every stage, suggesting novel lncRNAs could preferentially be involved in stage-specific functions. Specific expression is defined as an lncRNA expression of at least 3 FPKM by RNASeq data and at least 3-fold higher than those at the other two stages (*P* < 0.05). Under this definition, 171 (6.1%), 343 (12.3%) and 735 (26.3%) of novel lncRNAs and 4005 (3.6%), 1631 (1.5%) and 4098 (3.7%) of annotated lncRNAs were identified to be specifically expressed in Spga, Spcy and Sptd, respectively. Stage-specific lncRNAs were indicated under the stage-specific expression column in GermlncRNA.

However, we did not observe a large difference from the SAGE data. In total, 31.3% of annotated and 29.1% of novel lncRNAs were supported by at least one SAGE tag. At each stage, 4.7–4.9% of annotated and 3.9–4.3% of novel lncRNAs were abundant (tag count ≥ 5) (Table 1). We did not identify any correlation between lncRNAs expression and the type of lncRNA.

### Association with regulatory features

**Figure 5.**
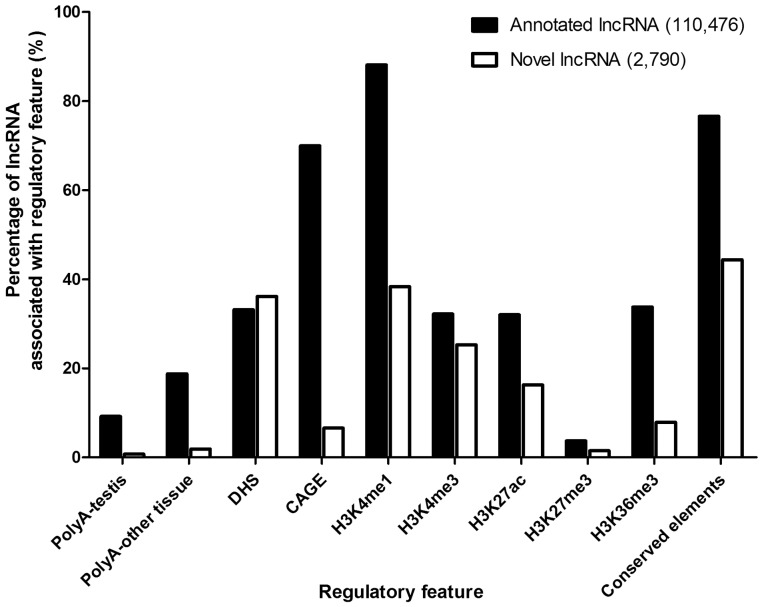
Percentages of annotated and novel lncRNAs associated with various regulatory features.

To elucidate the potential regulations associated with lncRNAs, the presence of regulatory features (Table 2, Figure 5) was examined, including (i) Transcription evidence: polyadenylation (PolyA) and CAGE signals for 3′- and 5′-terminals of a transcript, respectively; (ii) Chromatin-related features: DHS and five types of histone modifications, including H3K4me1, H3K4me3, H3K27ac, H3K27me3 and H3K36me3. These features are commonly associated with either promoter or enhancer (Table 2) and provide insights into possible regulation of lncRNA expression and (iii) Conserved elements: PhastCons mammals conserved elements (30-way multiz alignment) that mark evolutionary conserved regions among placental mammalian genomes predicted by phylogenetic hidden Markov model (phylo-HMM) (49).

To examine the difference and similarity between annotated and novel lncRNAs, we compared the feature association rates between them. For transcription features, a significantly higher percentage of annotated lncRNAs was found to accommodate PolyA (9.1 vs. 0.8%; *P* < 0.001 for testis, 18.8 vs. 1.9%; *P* < 0.001) for other tissues) or CAGE (70.0 vs. 6.6%; *P* < 0.001) signals when compared with novel lncRNAs (Figure 5). For chromatin-related features, DHS was associated with roughly similar proportions of annotated and novel lncRNAs (33.1 vs. 36.1%; *P* = 0.001), indicating that they are comparably accessible by DNA-binding proteins (Figure 5). The associations of two enhancer-associated features, H3K4me1 (88.1 vs. 38.4%; *P* < 0.001) and H3K36me3 (33.7 vs. 7.9%; *P* < 0.001), were significantly higher for annotated lncRNAs (Figure 5), indicating a smaller proportion of novel lncRNAs were enhancer-associated. The annotated lncRNAs also showed a higher association for active promoter features, H3K4me3 (32.2 vs. 25.3%; *P* < 0.001) and H3K27ac (32.0 vs. 16.3%; *P* < 0.001). We did not find high percentage of repressive mark, H3K27me3, in both annotated and novel lncRNAs (3.7 vs. 1.5%; *P* < 0.001), which indicates most lncRNAs do not have repressed promoters. Lastly, there was a larger proportion of annotated lncRNAs associated with conserved elements (76.6 vs. 44.4%; *P* < 0.001) (Figure 5), suggesting that novel lncRNAs were generally less conserved in mammals.

### Function prediction of novel lncRNAs

One of the major challenges in lncRNA study is function prediction. While the mode of regulation for lncRNAs is less defined, it was hypothesized that lncRNAs regulate cellular functions through interacting with coding genes (50, 51). For example, recent studies have showed that some lncRNAs involved in regulating expression of neighbour genes in *cis* (52, 53), while others were reported to regulate expression of coding gene targets on different chromosomes in *trans* (54, 55). To delineate functional regulation of the novel lncRNAs, the association between novel lncRNAs and the potential coding gene targets were examined. In GermlncRNA, protein-coding genes that are close genomic neighbours to novel lncRNAs in either direction were classified as *cis*-acting, and those that have significant similarity (at least 85% within 200 bp) in primary sequence to novel lncRNAs were classified as *trans*-acting. We hypothesized the potential functions of *cis*- and *trans*-acting lncRNAs were related to the coding partners, and therefore functional prediction of the coding partners by gene ontology was provided to illustrate the molecular function, biological process and cellular component involved. Such information can be retrieved by clicking on the corresponding coding gene target(s) in the search result table. In summary, 22.6% of *cis*-associated targets are located within 10 kb to the lncRNAs. On the other hand, 43% of the 2790 novel lncRNAs are *trans*-associated with at least one coding gene target, with an average length of 374 bp on the homologous region.

Other than coding gene interactions, non-coding RNA interactions with microRNAs have also been reported in post-transcriptional regulation (56, 57). MicroRNAs are short RNAs that associate with RNA-induced silencing complex (RISC) to inhibit gene expression. The complex binds to MREs at 3′-UTR (untranslated region) of target mRNA. Due to sequence similarity, some lncRNAs may rescue target gene expression that share similar MREs through competitive miRNA/RISC complex binding. In GermlncRNA, microRNAs targets of the novel lncRNAs were predicted by PITA algorithm (46) and listed in a table for microRNA targets. Each lncRNA was predicted to contain 388 miRNAs recognition elements (MREs) on average, with an average free energy change (ΔΔG) of −13.5.

For example, GlncRNA11284d was predicted to be a potential *cis*-acting lncRNA with two coding gene neighbours Rad18 and Oxtr, which are 9.9 kb upstream and 120 kb downstream to the lncRNA, respectively. A total of 182 miRNAs across 202 MREs, with an average **ΔΔ**G of −12.4 were identified. Gene ontology analysis suggested these two targets are involved in spermatogenesis and sperm ejaculation processes, respectively. The findings were supported by recent studies that Rad18 is critical in maintaining genomic stability in germ cell (58); while Oxtr involves in cAMP regulation in vas deferens epithelial cells (59). Taken together, the results suggested this *cis-*acting lncRNA may involve in various regulatory networks in male germ cell development.

## Discussion

lncRNAs have been demonstrated to be critical regulators in normal and disease development (10–15). Compared with protein-coding genes, lncRNA expressions are highly tissue- or cell-specific (29, 60, 61). This implies that lncRNAs expressed in specialized or rare cell types will be missed by public lncRNA annotation resources if the data sources do not contain the corresponding transcriptome information. The incomplete lncRNAs dataset will compromise research findings. To this end, we developed GermlncRNA to provide the first public online resource to search, visualize, download and analyse mouse male germ cell-specific lncRNAs.

To define annotated lncRNAs, we applied five public database resources for annotation. NONCODE contains the most lncRNA entries (67 595), followed by Ensembl (36 229), fRNAdb (30 335), UCSC (10 041) and Refseq (1279). It is interesting to note that there are significant differences on lncRNA annotation among these resources (Supplementary Figure S4). The difference could be attributed to the assembly algorithm and transcriptome datasets applied. Therefore, the quality and quantity of lncRNAs predicted will be highly variable. Even with the comprehensive annotation coverage in our analysis, we expected many germ cell specific lncRNAs would be missed. This is because the public resources did not include transcriptomes of specific male germ cell stages in the annotation assembly pipeline. The male germ cell information is mainly derived from testis transcriptome data. Unique RNA transcripts from minority male germ cell populations will be under-represented or undetected.

With the aid of bioinformatic prediction and male germ cell transcriptomic evidence, we were able to identify 2790 intergenic lncRNAs by HTA. Although we were also able to identify other novel gene associated lncRNAs, we did not include them in the current version due to potential ambiguity issues such as potential alternative splicing isoform of a coding gene. In any case, these novel germ cell specific lncRNAs are worthy of further follow-up, and those demonstrating stage-specific expression patterns may contribute to the stage-specific cellular functions, such as proliferation, transcriptional regulation of meiotic and early haploid stages of spermatogenesis. To allow a better understanding on these novel lncRNA candidates, we have included function prediction information by identifying the potential *cis*- and *trans*-acting coding gene targets. Although this approach may not represent all possible lncRNA regulation, we believe it is a sensible beginning approach. Some lncRNAs, such as *Xist* and *Air,* have been reported to regulate adjacent genes (52, 53); while others like *1/2sbsRNA*s and *BACE1-AS*, interact with target mRNAs of complementary sequence to promote or inhibit degradation (54, 55). Recent reports also suggested lncRNA could function as a competing endogenous RNA (ceRNA), which reduces the inhibitory effects of miRNAs by direct binding, such as *PTENP1* and *LincRNA-RoR* (19, 57, 62, 63). We will keep implementing data updates and functions to reflect new lncRNA regulation. For example, we envision including human male germ cell transcriptomes, mouse epigenome and direct lncRNA-protein interaction data in the upcoming release. Information on the recent and upcoming updates can be found on the GermlncRNA website.

In conclusion, GermlncRNA provides a systematic, integrative, user-friendly and easy-to-use platform to investigate lncRNAs involved in male germ cell development. Users can query for and clearly retrieve lncRNA of their interest through a variety of search options. Therefore, we believe that GermlncRNA can substantially improve the understanding on lncRNA biology in spermatogenesis.

## Funding

This work was supported by funds from the General Research Fund (GRF) from Hong Kong Research Grant Council (Grant numbers 469711 and 468312) and CUHK Direct Grants (MD10783 and MD11449), Faculty of Medicine, The Chinese University of Hong Kong and the Division of Intramural Research of the National Institute of Child Health and Human Development.

## Supplementary Data

Supplementary data are available at *Database* Online.

*Conflict of interest*. None declared.

Supplementary Data
