# Supplementary material for: GermlncRNA: a unique catalogue of long non-coding RNAs and associated regulations in male germ cell development
Source: Database (Oxford). 2015 May 16;2015:bav044. doi: 10.1093/database/bav044 (PMC4433719; doi:10.1093/database/bav044)
Supplement: Supplementary Data [file supp_2015_bav044_index.html]

Supplementary Data 

# GermlncRNA: a unique catalogue of long non-coding RNAs and associated regulations in male germ cell development

## Supplementary Data

files

- Supplementary Data - zip file
