# Supplementary material for: GermlncRNA: a unique catalogue of long non-coding RNAs and associated regulations in male germ cell development
Source: Database (Oxford). 2015 May 16;2015:bav044. doi: 10.1093/database/bav044 (PMC4433719; doi:10.1093/database/bav044)
Supplement: Supplementary Data [file supp_bav044_supp_data.zip › New Microsoft Office Word Document.docx]

**Supplementary Figure S1.** GermlncRNA schema.

**Supplementary Figure S2.** Schematic diagram showing bioinformatics pipeline of Hybrid Transcriptome Assembly (HTA).

**Supplementary Figure S3.** GermlncRNA search data panel overview.

**Supplementary Figure S4.** Venn diagram of annotated lncRNAs from five public databases.
