# Supplementary figures and images for: GermlncRNA: a unique catalogue of long non-coding RNAs and associated regulations in male germ cell development
Source: Database (Oxford). 2015 May 16;2015:bav044. doi: 10.1093/database/bav044 (PMC4433719; doi:10.1093/database/bav044)

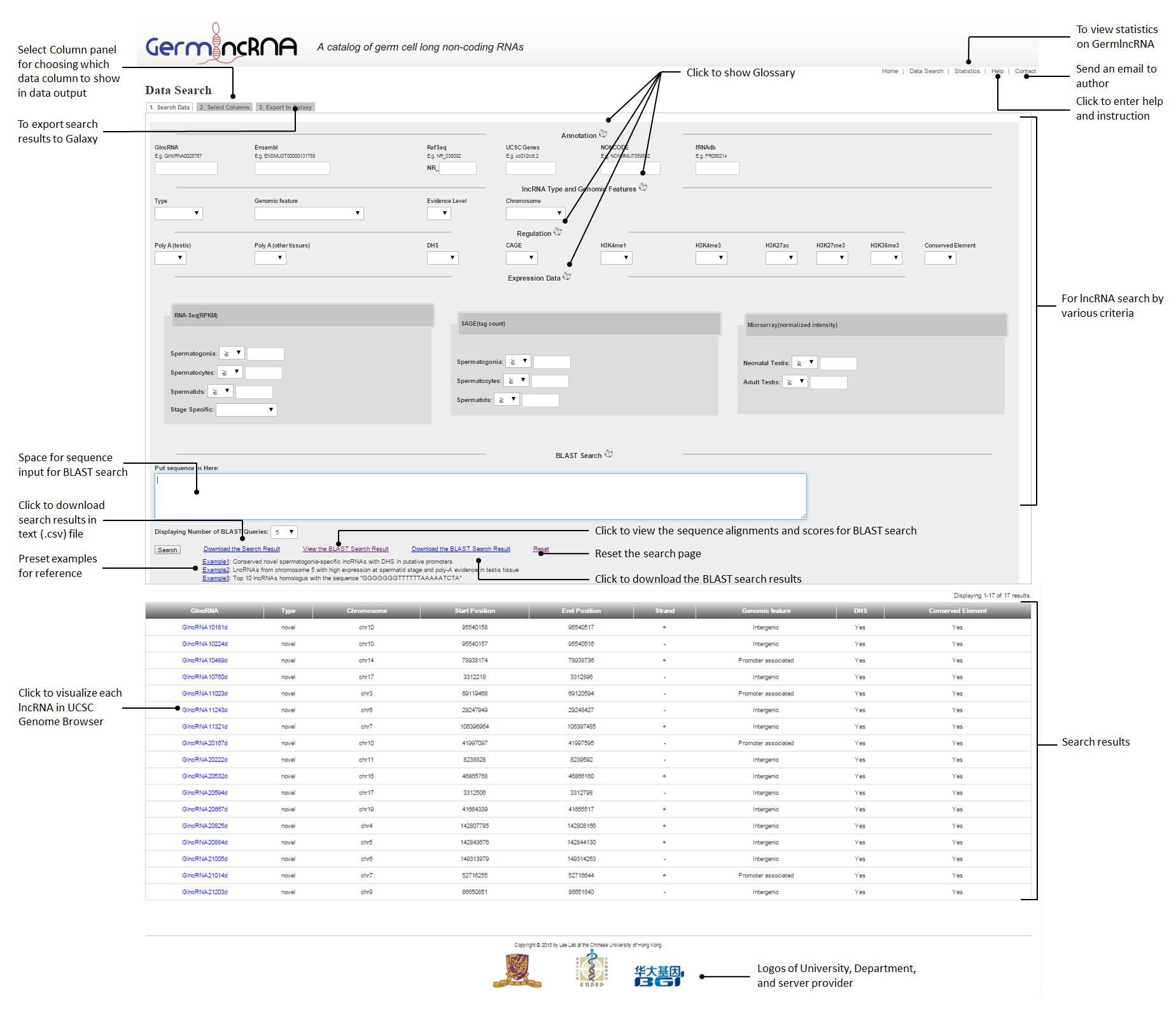

Supplement: Supplementary Data [file supp_bav044_supp_data.zip › Figure S3 Screenshot Data Search Revision 1.jpg]

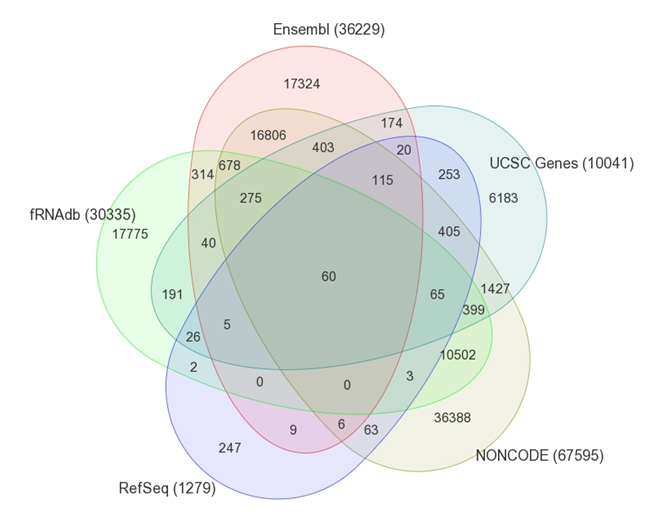

Supplement: Supplementary Data [file supp_bav044_supp_data.zip › Figure S4 Venn diagram_source db.jpg]

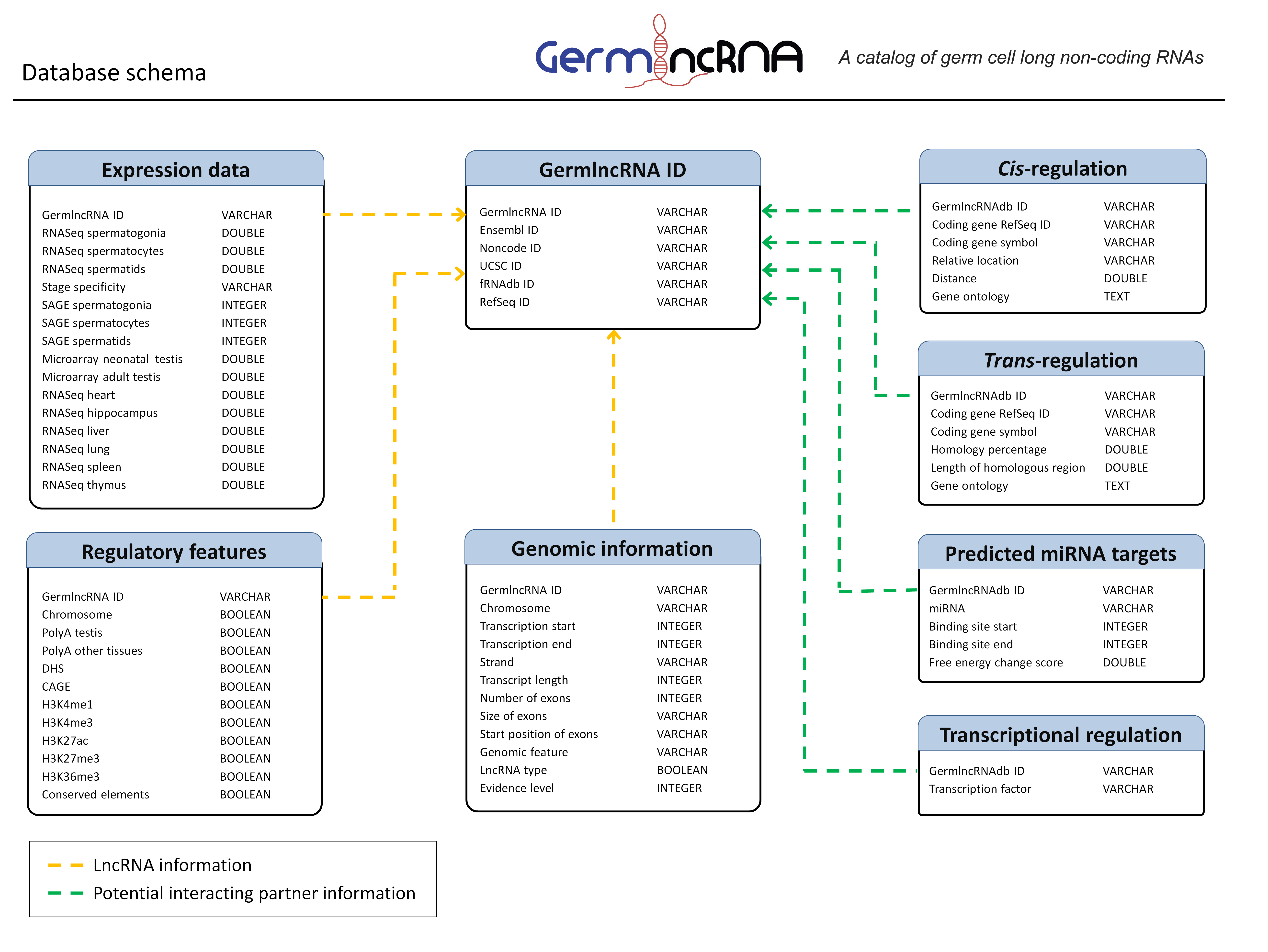

Supplement: Supplementary Data [file supp_bav044_supp_data.zip › Figure S1 GermlncRNA Schema_Revision 1.jpg]

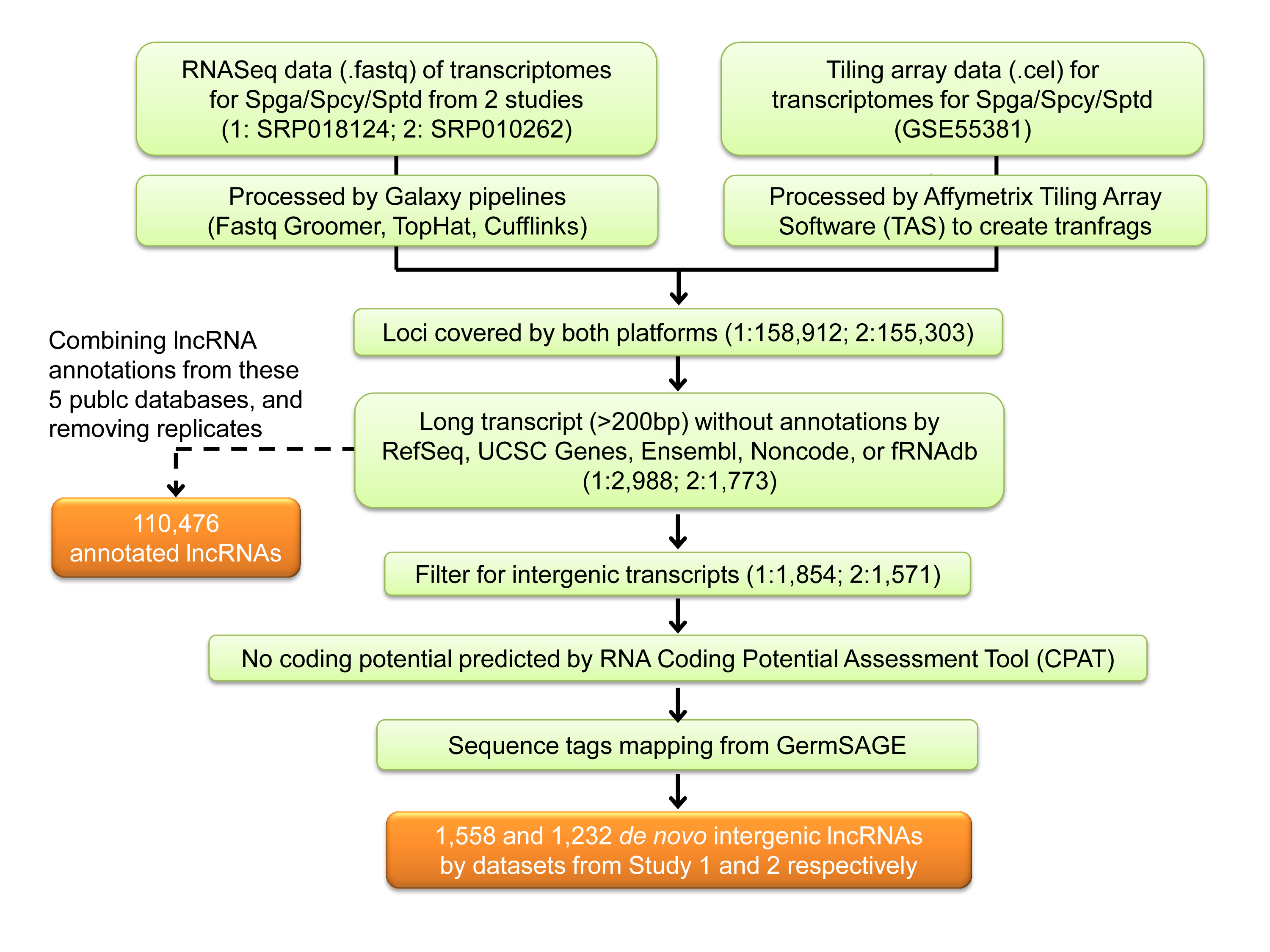

Supplement: Supplementary Data [file supp_bav044_supp_data.zip › Figure S2 HTA.jpg]
